# Yield Potential of Sugar Beet – Have We Hit the Ceiling?

**DOI:** 10.3389/fpls.2018.00289

**Published:** 2018-03-05

**Authors:** Christa M. Hoffmann, Christine Kenter

**Affiliations:** Institute of Sugar Beet Research, University of Göttingen, Göttingen, Germany

**Keywords:** sugar beet, yield potential, assimilate partitioning, sink limitation, water supply, storage losses, cambial rings

## Abstract

The yield of sugar beet has continuously increased in the past decades. The question arises, whether this progress will continue in the future. A key factor for increasing yield potential of the crop is breeding progress. It was related to a shift in assimilate partitioning in the plant toward more storage carbohydrates (sucrose), whereas structural carbohydrates (leaves, cell wall compounds) unintendedly declined. The yield potential of sugar beet was estimated at 24 t sugar ha^-1^. For maximum yield, sufficient growth factors have to be available and the crop has to be able to fully utilize them. In sugar beet, limitations result from the lacking coincidence of maximum irradiation rates and full canopy cover, sink strength for carbon assimilation and high water demand, which cannot be met by rainfall alone. After harvest, sugar losses during storage occur. The paper discusses options for a further increase in yield potential, like autumn sowing of sugar beet, increasing sink strength and related constraints. It is prospected that yield increase by further widening the ratio of storage and structural carbohydrates will come to its natural limit as a certain cell wall stability is necessary. New challenges caused by climate change and by prolonged processing campaigns will occur. Thus breeding for improved pathogen resistance and storage properties will be even more important for successful sugar beet production than a further increase in yield potential itself.

## Yield Increase in the Past

A high yield potential of agricultural crops is crucial for an efficient use of the available arable land. Yield potential is defined as the yield of a genotype grown in an environment to which it is adapted, without any limitations in water or nutrients or damage by pests, diseases, weeds, or other stresses (Evans and Fischer, 1999). For sugar beet, the yield potential has not been analyzed yet. In the past decades, sugar beet varieties have shown an annual increase in sugar yield by 1.5% (Märländer et al., 2003; Jaggard et al., 2010). This was partly due to increased spring temperature (Jaggard et al., 2007) and improved management practices. About 50% of the increase in yield and quality (0.9% a^-1^ for sugar yield) were achieved by breeding progress (Hoffmann and Loel, 2015), reflecting an increase of the yield potential.

When a high yield level has been achieved, breeding progress is essential for future yield improvements because increases achieved by improving technology, e.g., optimizing fertilizer use and crop protection, cannot be repeated (Jaggard et al., 2010). This begs the question about the extent to which breeding can increase the yield potential and which effect might arise from the expected climate change. The aim of the paper is thus to point out the perspectives for further improvement of sugar beet yield by analyzing the genetic potential as well as limiting factors apart from the effects of pests and disease.

## Shift in Assimilate Partitioning

Presuming that weather conditions cannot be changed, the genetic potential of a crop is the key factor for the potential yield. In order to assess whether the observed yield increase of sugar beet varieties will progress in the future, its physiological basis has to be analyzed. For this purpose, Loel et al. (2014) compared 17 old and new varieties. They found that the speed of leaf formation and the number of expanded cambial rings in the storage root had not changed in the registration period from 1964 to 2003. Hence, the cause of yield progress is neither increasing light interception and source activity (leaves), nor rising sink capacity (storage root).

**FIGURE 1:**
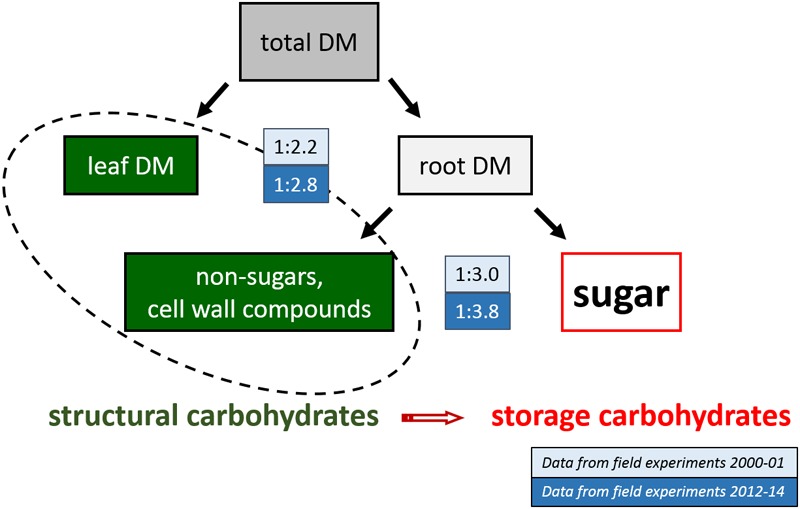
Shift in assimilate partitioning in sugar beet due to breeding progress based on data from 27 field experiments in Germany, 2000–2001 and six field experiments with three sowing dates and two varieties in 2012–2014. DM, dry matter.

Instead, breeding has obviously shifted assimilate partitioning within the plant (**Figure 1**). The total biomass produced by a sugar beet plant is partitioned into root and leaf dry matter (DM). The root DM consists mainly of sugar, which is targeted by breeding, and of all the non-sugar compounds including the molassigenic substances (mainly K, Na, amino acids) and the cell wall compounds (the marc, which forms the beet pulp) (Hoffmann et al., 2005; Hoffmann, 2010a). The ratios of marc to sugar found in experiments from 2000 to 2002 and from 2012 to 2014 clearly show a general shift toward less structural carbohydrates (leaves, cell wall compounds) and more storage carbohydrates (sugar). Hence, sugar yield was evidently increased on the expense of leaf dry matter and cell wall compounds in the storage root, so that the marc content of sugar beet varieties today is much lower than in the past (Hoffmann et al., 2005; Kenter and Hoffmann, 2009).

This process has continued further and marc content of current varieties is often below 4% (Hoffmann and Schnepel, 2016), whereas the sugar content did not change noticeably. Sugar and marc content of sugar beet varieties are nevertheless always closely related, which can be explained by an optimal cell volume for sugar storage (Milford, 1973; Kenter and Hoffmann, 2009). Hoffmann (2010a) confirmed a linear relation between sugar and marc in two sets of sugar beet varieties tested in the 1980ies and in 2006, but the regression line had shifted toward a lower marc:sugar ratio and a lower level of marc content in the more recent varieties.

This change in dry matter composition caused by a shift in assimilate partitioning is an unintended side-effect of the breeding progress so far. It is not clear yet, whether it forms the functional basis for the increase in yield potential and will therefore continue with further yield increase. Moreover, the question arises at which point the reduction in cell wall compounds will become limiting.

## Estimation of the Yield Potential

In the absence of pests and diseases, the realization of the genetic potential of sugar beet depends on the regional weather conditions. The growth of the storage root of sugar beet has no specific growth stages and accordingly no phase of maturation (Meier et al., 1993). Consequently, sugar yield increases with the length of the growing period, i.e., the number of days between sowing and harvest, and thus intercepted radiation (Scott et al., 1973). From this relation, the yield potential can be estimated.

Assuming average weather conditions, early sowing and late harvest, the maximum light interception of sugar beet is about 2,200 MJ photosynthetic active radiation (PAR) per ha during the growing season in Germany (Hoffmann and Kluge-Severin, 2010). The intercepted light is converted into biomass at 1.4 g DM per MJ PAR [radiation use efficiency (RUE); Monteith, 1977; Hoffmann and Kluge-Severin, 2010] to 1.8 g DM per MJ PAR (Werker and Jaggard, 1998; Qi et al., 2005). Assuming a high radiation interception (2,200 MJ ha^-1^) and very optimistic RUE (2.2 g MJ^-1^), potential sugar yield is 24 t ha^-1^. This value is close to earlier results by De Wit (1967) based on theoretical assumptions for assimilation and weather conditions.

Kenter et al. (2006) used data from a large series of field trials to calculate the potential yield of sugar beet. The maximum growth rates, which sugar beet had achieved under various environmental conditions in Germany, were summed up over the growing period. According to this calculation, the potential yield of sugar beet is 42 t of total DM ha^-1^ with 24 t of sugar ha^-1^, i.e., the different approaches give the same result.

## Utilization of Growth Factors

### Light Interception

A prerequisite for high yields is the coincidence of complete canopy cover with periods of high radiation in spring/early summer (Scott and Jaggard, 1978). It is thus expected that the cultivation of autumn sown beet could greatly increase yield by better synchronization of irradiance and canopy cover (Jaggard and Werker, 1999; Hoffmann and Kluge-Severin, 2010, 2011). Currently, autumn sowing of sugar beet is restricted because of lacking bolting resistance, but also, and probably of similar importance, because of the insufficient frost hardiness of sugar beet (Kirchhoff et al., 2012; Reinsdorf and Koch, 2013; Loel and Hoffmann, 2014, 2015). Therefore, the yield benefit from autumn sown beets can only be calculated theoretically (Jaggard and Werker, 1999; Hoffmann and Kluge-Severin, 2011). To get more knowledge about the maximum yield of long growing sugar beet, Schnepel and Hoffmann (2016a) conducted a pot experiment in the greenhouse where vernalisation was prevented. The sugar yield increased continuously with time, reaching about 500 g of sugar per plant after 800 days of growing. A crop with 100,000 plants per ha could thus obtain around 50 t of sugar per ha which can basically be assumed as the potential yield of sugar beet. However, this will not be possible within one growing season. It is a question of efficiency whether one crop produces more biomass in a prolonged growing period than subsequent crops in a rotation, even when risks of pests and diseases are neglected.

Within the current system of cultivation, an early sowing date is essential for high yield. To fully benefit from early spring sowing, the plants have to emerge quickly even under low temperature conditions. Furthermore, the crop has to accelerate canopy closure compared to plants sown at the normal date. Therefore, adapted sugar beet varieties need to have a lower minimum temperature for emergence and leaf formation (<3°C; Milford et al., 1985) and higher growth rates at temperatures below 10°C. Furthermore, the vernalisation requirement should be higher and the bolting sensitivity of the varieties lower than today (Milford et al., 2010) to ensure yield formation in a vegetative phase. Therefore, in future it will be important to select for sugar beet varieties, which are adapted in their yield formation process to low temperatures (high cold tolerance and bolting resistance).

### CO_2_ Assimilation

It has been demonstrated that rising atmospheric CO_2_ concentrations enhance sugar beet growth through higher assimilation rates (Demmers-Derks et al., 1998; Manderscheid et al., 2010). Yield increase in the past can thus partly be attributed to increasing CO_2_ in the atmosphere. However, Manderscheid et al. (2010) showed that white sugar yield increased by only 10–15%, when CO_2_ concentration was elevated from 375 to 550 ppm as forecasted for the middle of the 21^st^ century, and thus less than expected from theory. This low response provides evidence for a sink limitation of beet growth.

Sink limitation (except for the phase of incomplete canopy closure in late spring) is further emphasized by results obtained under drought stress (Mäck and Hoffmann, 2006), where sugar accumulated in the leaves, resulting in a feed-back inhibition to assimilation, presumably due to lacking storage capacity in the root (Hoffmann, 2010b, 2014). Also Schnepel and Hoffmann (2016a) observed a decline in the rate of photosynthesis with increasing sugar concentration in the beet. It can thus be concluded that for a further increase in yield potential and to fully exploit rising atmospheric CO_2_, in particular the sink capacity of sugar beet has to be enhanced.

## Limitations – Actual Yield

The actual yield is always lower than the potential one, because weather conditions are usually not optimal and management operations, headlands and many other factors restrict sugar beet yield in commercial fields (Trimpler et al., 2017). The gap between attainable yield measured in official variety testings and the actual yield at farmers’ fields amounts to more than 30% in some countries (Jaggard et al., 2012). In the following, some important physiological factors are discussed which will limit the actual sugar beet yield in future.

### Water Supply

Even in years with favorable conditions for sugar beet growth, water shortage may occur during the summer months, when ambient temperature, water saturation deficit and thus transpiration demand are high. Therefore, yield reductions resulting from water shortage generally occur, in particular on light soils, and climate change is expected to fortify this effect in the future (Schindler et al., 2007; Okom et al., 2017).

With increasing level of yield and dry matter production, the probability of water limitation will increase as well. Assuming a transpiration coefficient of about 200 L of water to produce 1 kg of dry matter (Ehlers and Goss, 2003; Hoffmann, 2014), the production of 24 t of sugar ha^-1^, which equals a total DM of about 42 t ha^-1^ and a root DM of 31 t ha^-1^, will require more than 8.000 L water (800 mm). In the traditional sugar beet cultivation areas, this demand can hardly be met by rainfall alone and consequently, yield formation will always be restricted to a certain extent. Without additional water supply by irrigation, the potential yield can never be approached, in particular not if breeding achieves further increase and in a changing climate.

Therefore, varieties are needed, which are drought tolerant and respond with a lower yield reduction to insufficient water supply, not only to secure higher yields, but also for a higher yield stability under various environmental conditions. The water demand can be reduced when less DM is partitioned into leaf DM, leaving a higher percentage of assimilates for the storage of sugar. In pot experiments it has been shown that sugar beet can achieve very high yields with a much lower leaf DM than is usually produced in the field (Hoffmann, 2014). Furthermore, a smaller leaf apparatus was associated with lower transpiration rates at constant yield (Hoffmann, 2014). A possible way to increase water use efficiency and produce more DM from the available water (“more crop per drop”; Blum, 2005, 2009) could thus be the reduction of the often luxurious canopy of sugar beet, but this might conflict with the aim to accelerate early leaf growth and might also increase weed competition.

### Storage Losses

**FIGURE 2:**
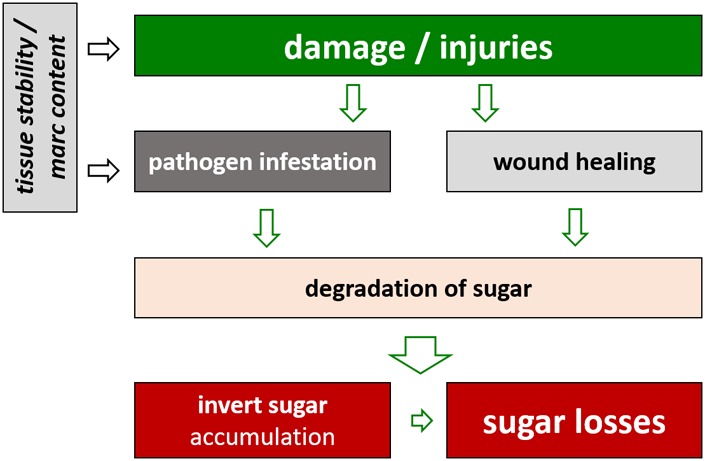
Effect of damages of sugar beet roots on sugar losses during storage.

Apart from limitations due to unfavorable weather conditions, further reductions in harvested sugar yield occur before the roots are processed in the factory. During storage sugar is cleaved to provide energy for life-sustaining processes of the sugar beet plant (Klotz and Finger, 2004). As the processing campaigns in the sugar factories are currently being extended, varieties are needed which can retain the assimilated sugar during the storage period. Among other factors such as damage during harvest operations, the genotype has an effect on the storability of sugar beet (Schnepel and Hoffmann, 2016b). Interestingly, there is evidence that varieties with lower marc content show higher sugar losses and invert sugar accumulation during storage. This seems to be the consequence of a higher susceptibility toward damage during harvest operations and toward the subsequent infestation with mold and rots during storage (**Figure 2**; Hoffmann and Schnepel, 2016; Schnepel and Hoffmann, 2016b).

## Hit the Ceiling?

The essential question concerning future progress is whether the success of continuously increasing the yield level can be continued. If a further increase in potential sugar yield will be related to a further decline in cell wall compounds (marc content), problems will probably arise. The proportion of cell wall compounds will approach a natural limit, because a certain cell wall stability is needed to counterbalance the turgor pressure in the cell, but also to form a barrier to mechanical strain as well as pathogen attacks. As marc content and root yield are usually negatively correlated (Hoffmann et al., 2005), varieties with the highest yield will most likely not show best storability. Hence, for efficient sugar beet production in the future, a differentiation between genotypes with either highest yield potential or best storage properties will be necessary.

A further increase in yield potential will probably be based on rising root yield, as seen in the past (Märländer et al., 2003, 2017), while increasing sugar content is not very likely to contribute largely to higher sugar yields. The uptake of sucrose to parenchymal cells in the storage root results from the membrane transport of solutes. As it is inhibited by increasing cell turgor as determinant of sink strength because of the inhibition of the plasma membrane ATPase (Wyse et al., 1986), the increase of the sugar content is limited.

Greenhouse experiments have shown that sugar beet plants can obtain very high storage root yields with little leaf dry matter (Hoffmann, 2010b; Schnepel and Hoffmann, 2016a). As plants usually feature a higher leaf area index than required for assimilation, a reduction of the leaf dry matter after canopy closure might contribute to a further improvement of root yield. But, this shift in assimilate partitioning also requires an increased sink strength of the storage root.

Milford (1973) hypothesized that a possible way to increase sink strength would be a higher number and the complete development of all cambial rings in the storage root. However, sugar beet with an extended growing period (>300 days) neither formed a higher number of cambial rings nor became the outer cambial rings fully developed (Schnepel and Hoffmann, 2016a). Root yield constantly increased due to the development of the inner 5 to 6 rings as also found by Loel et al. (2014) in the comparison of old and new varieties. This is underlined by transcript analyses by Bellin et al. (2007), who reported a spatial gradient from the inner to the outer root zone in sugar beet. Cells in the outer cambial rings remained small and undeveloped, so that mature beets simultaneously contained transcripts typical of innermost sucrose-rich cells and of differentiating sucrose-poor cells in the outer parts. Hence, in contrast to former assumptions, the sink strength of the storage root of sugar beet seems not to be determined solely by cambial ring formation.

As there is currently no strategy available to increase sink strength in sugar beet, efforts should focus on exploiting the full potential of about 24 t ha^-1^ of sugar, which current varieties have already achieved in single field trials (IfZ, unpublished data). Due to the aforementioned limitations for yield formation, this cannot be realized in all environments, but there is still potential for further agronomic improvement. Laidig et al. (2014) demonstrated that in addition to the genetic improvement and in contrast to other crops, sugar beet shows a high increase in agronomic performance.

Nevertheless, future challenges will grow. Climate change will not only increase the risk of drought stress, but also the infestation pressure of pests and diseases (Juroszek and von Tiedemann, 2013; Kremer et al., 2016). Moreover, the availability of crop protection active ingredients is decreasing due to resistance development (Varrelmann and Märländer, 2017) and restrictive approval practices. In addition, prolonged processing campaigns will cause losses by both earlier harvest and longer storage periods (Kenter and Hoffmann, 2007). Hence, in the future breeding is expected to contribute more to successful sugar beet growing by improving pathogen resistance and storage properties of the beet than by increasing the yield potential itself.

## Author Contributions

CH and CK: conceptualized the paper; CH: wrote the manuscript; CK: edited and completed it.

## Conflict of Interest Statement

The authors declare that the research was conducted in the absence of any commercial or financial relationships that could be construed as a potential conflict of interest.
